# Exploration of bacterial lipopolysaccharide-related genes signature based on T cells for predicting prognosis in colorectal cancer

**DOI:** 10.18632/aging.206041

**Published:** 2024-08-06

**Authors:** Lichao Cao, Ying Ba, Fang Chen, Shenrui Zhang, Hezi Zhang

**Affiliations:** 1 Shenzhen Nucleus Gene Technology Co., Ltd., Shenzhen, Guangdong, China; 2 Shenzhen Nucleus Huaxi Medical Laboratory, Shenzhen, Guangdong, China; 3 Shanghai Nucleus Biotechnology Co., Ltd., Shanghai, China

**Keywords:** colorectal cancer, microorganisms, lipopolysaccharide, tumor microenvironment, T cells

## Abstract

Purpose: The intratumoral microorganisms participates in the progression and immunotherapy of colorectal cancer (CRC). However, due to technical limitations, the impact of microorganisms on CRC has not been fully understood. Therefore, we conducted a systematic analysis of relationship between bacterial lipopolysaccharide (LPS)-associated genes and immune cells to explore new biomarkers for predicting the prognosis of CRC.

Methods: The single-cell RNA sequencing data and the Comparative Toxicogenomics Database were used to screen T cells-associated LPS-related genes (TALRGs). Then, we established and validated the TALRGs risk signature in The Cancer Genome Atlas Colon Adenocarcinoma (TCGA-COAD) cohort and GSE39582 cohort. Besides, we compared the differences in tumor-infiltrating immune cell types, immunotherapeutic response, somatic mutation profiles, and tumor mutation burden (TMB) between high-risk group and low-risk group. In addition, the immunotherapeutic cohort (Imvigor210) treated with an anti-PD-L1 agent was performed to explore the potential value of the TALRGs signature on immunotherapy.

Results: Five prognostic TALRGs were identified and selected to build the prognostic model. The high-risk group had poor prognosis in both TCGA-COAD cohort (*P* < 0.0001) and GSE39582 cohort (*P* = 0.00019). The areas under the curves (AUCs) of TALRGs signature were calculated (TCGA-COAD cohort: 0.624 at 1 years, 0.639 at 3 years, 0.648 at 5 years; anti-PD-L1 cohort was 0.59). The high-risk group had advanced pathological stages and higher TMN stages in both TCGA-COAD cohort and GSE39582 cohort. The high-risk group had the higher infiltration of immunosuppressive cells, the expressions of immune checkpoint molecules, the IC50 values of chemotherapy drugs, and TP53 mutation rate (*P* < 0.05). In addition, patients with high TMB had worse prognosis (*P* < 0.05). Furthermore, the Imvigor210 also showed patients with high-risk scores had poor prognosis (platinum-treated cohort: *P* = 0.0032; non-platinum-treated cohort: *P* = 0.00017).

Conclusions: Microorganisms are closely related to the tumor microenvironment to influence the progression and immune response of CRC via stimulating T cells through LPS-related genes. The TALRGs signature contributed to predict the prognosis and immunotherapy of CRC, and became new therapeutic targets and biomarkers of CRC.

## INTRODUCTION

Colorectal cancer (CRC) is the second leading cause of cancer-related death worldwide, with high morbidity and mortality [1]. With the development of economy, the incidence and death rate of CRC are gradually increasing, especially in individuals under 50 years of age [2]. Treatment options for CRC are complex, usually with surgery as the primary treatment, radiotherapy, chemotherapy, targeted therapy, and combination therapy as adjuvant treatments [3]. Unfortunately, not all patients benefit from these treatments, and there is also an increased rate of treatment failure due to high recurrence rates and resistance to anti-cancer drugs, which is closely associated with the heterogeneity of the CRC tumor microenvironment (TME) [4, 5]. Therefore, predicting the prognosis and determining the best treatment for poor prognosis of CRC from the perspective of TME is of great significance for improving the survival time and quality of patients.

The role of microorganisms in the occurrence, diagnosis, prognosis and treatment of cancer has been increasingly recognized. Some studies have found that the infections of gut microbiota may be associated with the development of CRC [6]. For example, a potential association between streptococcal infections and gastrointestinal tumors was discovered as early as 1950s [7]. In addition, gut and intratumoral microorganisms affected the immune infiltrating cells of TME via their derived metabolites, genotoxins, and antigens, thereby regulating the antitumor immune response [8, 9]. For instance, the immunogenic intestinal bacteria (*Helicobacter hepaticus*) inhibited tumor growth of colon adenocarcinoma (COAD) by activating CD4+ T cells- and B cells-associated anti-tumor immunity [10]. Bacterial lipopolysaccharides (LPS) are present in both cancer cells and immune cells of TME [11], which can bind to the TLR4 of monocytes, causing them to differentiate into an immunosuppressive M2 phenotype [12, 13]; and can also promote the recruitment of CD11b^+^Gr-1^+^ myeloid-derived suppressor cells (MDSCs) and CD1d^+^CD5^+^ regulatory B (Breg) cells on tumor cells, which together inhibit the local anti-tumor T cells response [14]. Actually, unlike gut microbes, the comprehensive characterization of intratumoral microorganisms’ signatures related to immune responses is still in its infancy due to technical limitations. Therefore, through immune infiltrating cells in TME, we can explore the role of major pathogenic components of intratumoral microorganisms on the occurrence, development, prognosis and treatment of cancer, laying a foundation for the clinical prevention and treatment of cancer.

In this study, we aim to identify a T cells-associated LPS-related gene (TALRG) signature as a biomarker for the diagnosis and prognosis of CRC. We first obtained differentially expressed genes (DEGs) in T cells from the single-cell RNA sequencing (scRNA-seq) data and LPS-related genes from the Comparative Toxicogenomics Database (CTD). Then, TALRGs were obtained by making the intersection of DEGs in T cells and LPS-related genes. Subsequently, Univariate and multivariate Cox analyses were used to identify a prognostic 5-TALRGs signature. This study revealed TALRGs’ clinical value in CRC and provided recommendations for finding new treatment options of CRC.

## METHODS

### Acquisition and processing of data

The scRNA-seq dataset GSE200997 was obtained from the Gene Expression Omnibus (GEO, https://www.ncbi.nlm.nih.gov) database, which included 16 CRC samples and 7 adjacent samples of normal colon tissue. There was a total of 49,859 cells in all samples, including 31,586 cells from tumor samples and 18,273 cells from normal samples. A total of 5286 LPS-related genes were downloaded from CTD database (http://ctdbase.org/).

The Cancer Genome Atlas Colon Adenocarcinoma (TCGA-COAD) cohort was downloaded from UCSC Xena platform (https://xenabrowser.net/datapages/), which is used to construct the prognosis model, including the mRNA expression data, clinical information (gender, age, microsatellite status, overall survival (OS), pathological stage, TNM stage), and mutation profiling data. The detailed information was shown in Supplementary Table 1. The validation dataset (GSE39582) was downloaded from the GEO database, and the detailed information was shown in Supplementary Table 2.

According to the published report [15], we gained an immunotherapeutic cohort (IMvigor210), which was treated with atezolizumab (anti-PD-L1 agent). Based on the guideline on http://research-pub.gene.com/IMvigor210CoreBiologies, the IMvigor210CoreBiologies R package was used to acquire Expression sets and clinical information of this cohort [16]. The samples were divided into platinum-treated cohort and non-platinum-treated cohort based on whether or not they received platinum-based chemotherapy.

### scRNA-seq dataset analysis and TALRGs identification

The cell filtration criteria in the scRNA-seq dataset (GSE200997) were consistent with previous study [17]. According to the previous report [17], the filtered data was used to reduce the dimensionality of the features. The cells were then clustered using the FindNeighbors and FindClusters functions. The t-distributed stochastic neighbor embedding, and uniform manifold approximation and projection (UMAP) algorithms were used to reduce the dimensionality and visualize clustering classification based on the first 20 principal components selected. Then, we used SingleR package of R software to annotate the cell types of clusters, with “HumanPrimaryCellAtlasData” as reference [18]. Subsequently, we identified DEGs between tumor and normal cells in T cells using the FindMarkers function with logfc.threshold = 0.585. Moreover, the Kyoto Encyclopedia of Genes and Genomes (KEGG) enrichment analyses of DEGs in T cells were conducted using the Database for Annotation, Visualization and Integrated Discovery (DAVID) database (https://david.ncifcrf.gov/conversion.jsp). Finally, we selected the intersection of DEGs in T cells and bacterial LPS-related genes to obtain TALRGs.

### Development and validation of the TALRGs prognostic model

TCGA-COAD cohort was used to recognize the prognostic TALRGs and established a prognostic risk model. Univariate Cox analysis was used to screen the prognostic signature using Survival R package. Kaplan–Meier analysis was performed to evaluate the relationship between mRNA expression of prognostic TALRGs and overall survival (OS). According to the each TALRG’ coefficients (which obtained from the multivariate Cox results), the risk score was calculated by the following formula [17]:


$$\begin{array}{l} risk\;score=\sum_{i=1}^{n}Cox\;coefficient\;of\;gene\;\chi i\;\;\times \\ \;\;\;\;\;\;\;scaled\;expression\;value\;of\;gene\;\chi i \end{array}$$

Kaplan–Meier analysis and time-dependent receiver operating characteristic (ROC) curve were applied to validate the predictive ability of the TALRGs prognostic model using the survminer R package and survivalROC R package, respectively. To further confirm the prediction model, we introduced a validation dataset: GSE39582. The prediction ability of the model was verified by the above method.

### Assessment of clinicopathological characteristics

TCGA-COAD cohort and GSE39582 dataset were performed to validate the prognostic capability of TALRGs prognostic model. According to the constructed risk scoring model, the dataset was divided into high-risk group and low-risk group. Kruskal-Wallis test and Wilcoxon test were used to investigate the relationship between TALRGs prognostic model and clinicopathological characteristics, including pathological stage and TNM stage.

### Establishment of nomogram

Nomogram is a multivariate regression analysis based on clinicopathological information and risk score. Based on the risk score and clinical factors such as age, sex, microsatellite status, and pathological stage, the nomogram was constructed using the TCGA-COAD cohort using rms R package. The calibration curve was employed to evaluate the suitability of clinical use.

### Estimation of immunological properties of TALRGs signature

Similar to the previous approach [16], we employed CIBERSORT algorithm to evaluate the infiltration of 22 types of tumor-infiltrating immune cells [19]. The Wilcoxon test was applied to compare to the proportions of immune cells between low- and high-risk groups (*P*-value < 0.05). Then, we employed unpaired *t*-test and Wilcoxon test to assess the difference of the mRNA levels of immune checkpoints and IC_50_ value of chemotherapy drugs between the two risk groups, respectively.

### Analysis of mutation characteristics of TALRGs signature

We employed the maftools R package to analyze and visualize the somatic mutation profiles. Subsequently, the correlation between the mutation status of gene and OS was evaluated. The value of tumor mutation burden (TMB) was calculated and visualized by the maftools R package. The Kruskal-Wallis test was performed to compare the TMB value between the two risk groups. The surv_cutpoint algorithm of survival R package was used to determine the optimal cutoff value of TMB, and then all samples were divided into high-TMB group and low-TMB group. Kaplan–Meier analysis was used to evaluate the relationship between TMB values and OS. *P* < 0.05 was considered statistically significant.

### The role of the TALRGs signature in immunotherapy

Similar to the previous study [16], we validated the constructed TALRGs signature using platinum-treated cohort and non-platinum-treated cohort, respectively. Secondly, patients with complete response (CR) or partial response (PR) were classified as responders and compared with non-responders (stable disease (SD) or progressive disease (PD)), and the risk score for each patient was calculated according to the built risk score model. Kaplan–Meier analysis and ROC curve were applied to assess predictive ability of the risk model. Finally, we employed the Wilcoxon test to compare the tumor mutation load and neoantigen load between the two risk groups.

## RESULTS

### scRNA-seq analysis of CRC and identification of TALRGs

**Figure 1:**
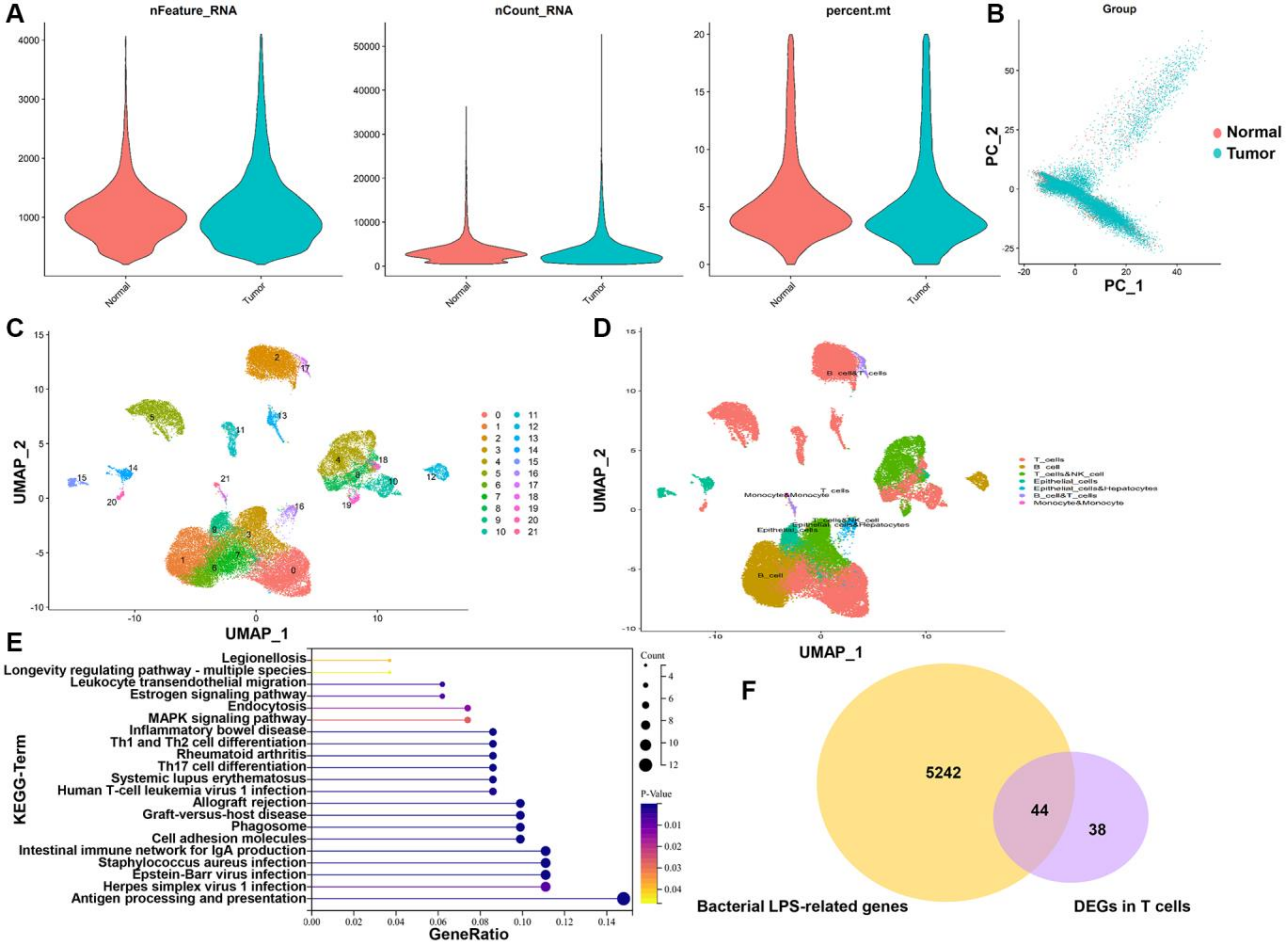
**TALRGs identification from scRNA-seq data and CTD.** (**A**) The quality control of scRNA-seq data. (**B**) The dimensionality of tumor cells and normal cells was reduced by PCA. (**C**) UMAP distribution of the 22 cell clusters. (**D**) The cell clusters were annotated into 6 main cell types. (**E**) KEGG analysis of DEGs in T cells. (**F**) The Venn diagram of DEGs in T cells and LPS-related genes.

In all, 47,958 high-quality cells were obtained after strict quality control (Figure 1A), including 30,341 tumor cells and 17,617 normal cells. The dimensionality reduction by principal component analysis (PCA) showed that tumor cells and normal cells were significantly different (Figure 1B). Subsequently, 22 distinct cell subgroups were identified and visualized by using UMAP method (Figure 1C). Through the annotation of 22 cell subgroups, a total of 6 major cell types were identified, mainly including T cells, B cells, NK cells, endothelial cells, hepatocytes, and monocytes (Figure 1D). Subsequently, we compared and analyzed the genes between tumor and normal cells in individual T cell clusters. T cells revealed 82 DEGs between tumor and normal cells (Supplementary Table 3). KEGG enrichment analysis showed that DEGs in T cells are mainly involved in “Antigen processing and presentation”, “Intestinal immune network for IgA production”, “Staphylococcus aureus infection” and “Cell adhesion molecules” (Figure 1E), which are correlative signaling pathways of bacterial LPS-related genes. A total of 5,286 bacterial LPS-related genes were obtained from the CTD database. Then LPS-related genes were overlapped with DEGs of T cells, and 44 TALRGs were obtained (Figure 1F).

### Establishment and validation of TALRGs signature

**Figure 2:**
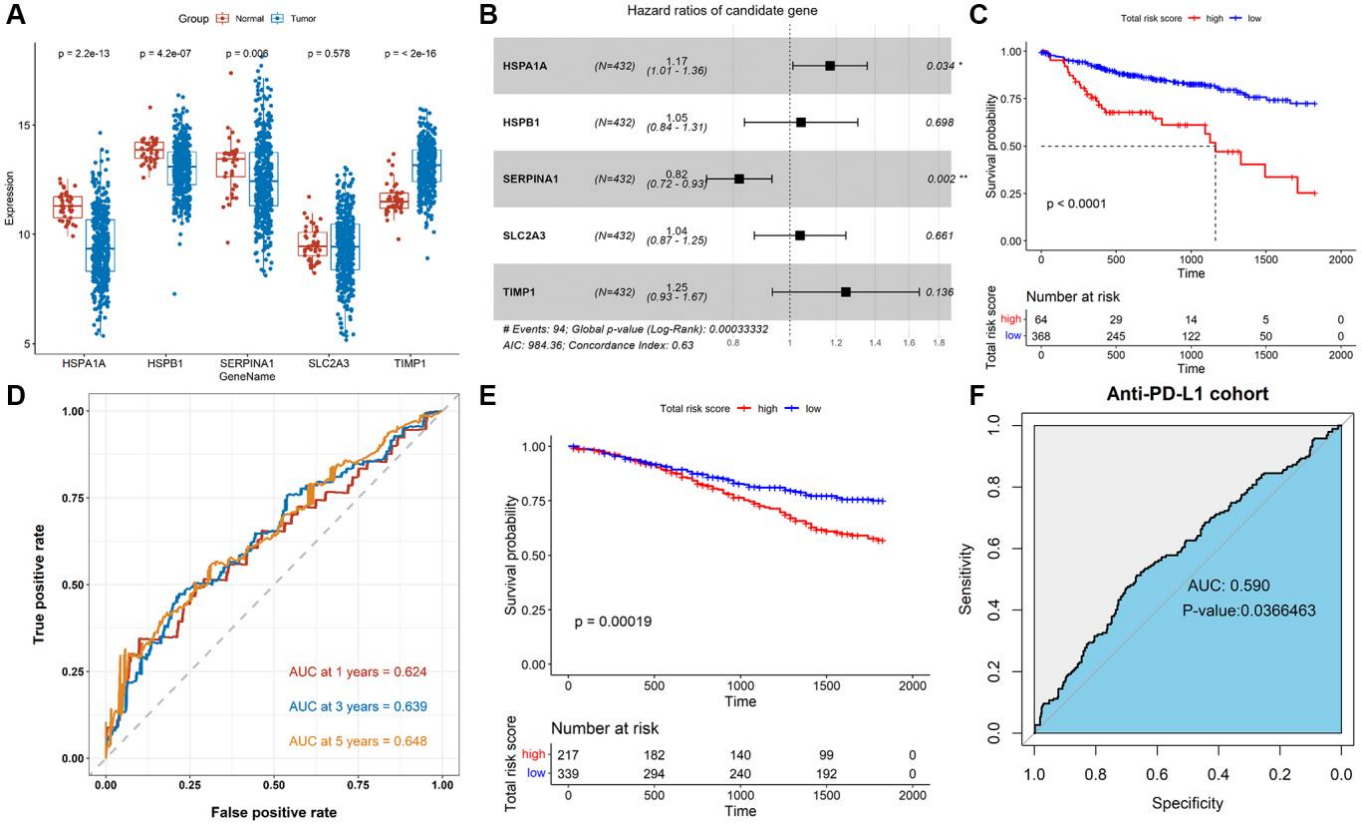
**Construction and validation of the TALRGs signature.** (**A**) Comparison of the TALRGs expression between tumor and normal samples. (**B**) The multivariate Cox analysis. (**C**) Kaplan–Meier analysis of the TALRGs signature in TCGA-COAD cohort. (**D**) ROC curve analysis of the TALRGs signature in TCGA-COAD cohort. (**E**) Kaplan–Meier analysis of the TALRGs signature in GSE39582 cohort. (**F**) ROC curve analysis of the TALRGs signature in anti-PD-L1 cohort.

We further studied the association of TALRGs with prognosis, and 5 optimal prognosis-related TALRGs with *P* < 0.05 were identified using univariate Cox analysis (Table 1), namely HSPA1A, HSPB1, SERPINA1, SLC2A3, and TIMP1. Compared with normal samples, the expressions of HSPA1A, HSPB1 and SERPINA1 were down-regulated in tumor samples, while TIMP1 was up-regulated in tumor samples. SLC2A3 expression had no significant difference between normal and tumor samples (Figure 2A). Furthermore, Kaplan–Meier survival analyses demonstrated that patients with increased HSPA1A (*P* = 0.018, Supplementary Figure 1A), SLC2A3 (*P* = 0.0073, Supplementary Figure 1D), and TIMP1 (*P* = 0.0031, Supplementary Figure 1E) showed poor survival, while patients with increased SERPINA1 indicated favorable survival (*P* = 0.02, Supplementary Figure 1C). There was no significant relationship between HSPB1 expression and OS (*P* = 0.28, Supplementary Figure 1B). Subsequently, multivariate Cox analysis established a TALRGs signature based on the TCGA-COAD cohort, and the hazard ratio of each gene was shown in Figure 2B. All patients were then divided into high- and low-risk groups based on the median risk score. The formula for the risk score model was as follows: risk score = (0.15872 × expression value of HSPA1A) + (0.04431 × expression value of HSPB1) + (−0.19961 × expression value of SERPINA1) + (0.0404 × expression value of SLC2A3) + (0.22075 × expression value of TIMP1). The Kaplan–Meier analysis manifested low-risk patients had a better survival advantage (*P* < 0.0001, Figure 2C). In addition, the area under the curve (AUC) of the TCGA-COAD cohort was 0.624 (at 1-years), 0.639 (at 3-years), and 0.648 (at 5-years), respectively (Figure 2D). The model was validated in the GSE39582 cohort. The OS rate of the low-risk group was higher than that of the high-risk group (*P* = 0.00019, Figure 2E). The AUC of anti-PD-L1 cohort was 0.59 with *P* = 0.0366463 (Figure 2F). These results suggested that TALRGs prognostic model is effective in predicting the prognosis of CRC patients.

**Table 1:** The detailed information on the identified prognostic TALRGs.

**TALRGs**	**HR**	**HR.95L**	**HR.95H**	***P*-value**
HSPA1A	1.234564836	1.089886942	1.398448111	0.0009216
HSPB1	1.233081483	1.030951791	1.474840973	0.02180952
SERPINA1	0.869881952	0.771966563	0.980216822	0.022142324
SLC2A3	1.173886946	1.020513968	1.350310341	0.024818267
TIMP1	1.304391157	1.060403191	1.604518267	0.011901692

### Evaluation of clinicopathological characteristics

**Figure 3:**
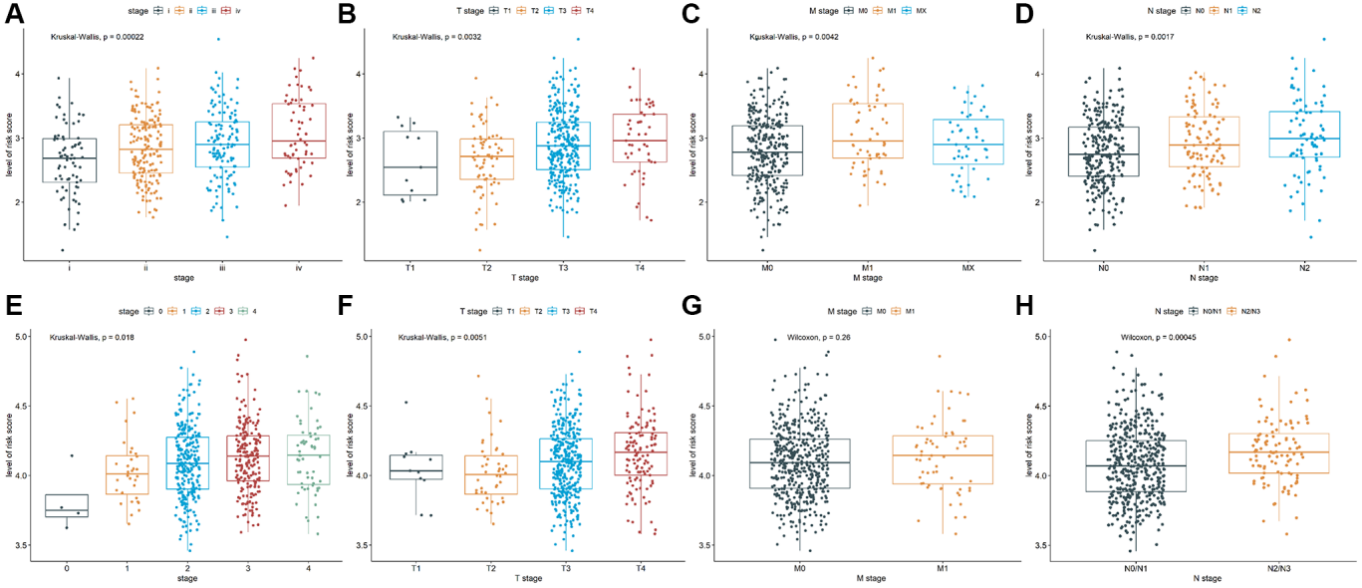
**Analysis of clinicopathological characteristics.** The Kruskal-Wallis test was used to analyze the relationship between risk score and (**A**) advanced pathological stages, (**B**) T stages, (**C**) M stages, and (**D**) N stages in TCGA-COAD cohort. The Kruskal-Wallis test was used to analyze the relationship between risk score and (**E**) advanced pathological stages and (**F**) T stages in GSE39582 cohort. The Wilcoxon test was performed to analyze the relationship between risk score and (**G**) M stages and (**H**) N stages in GSE39582 cohort.

To explore the relationship between prognostic values and clinicopathological characteristics of CRC patients, clinical traits in TCGA-COAD and GSE39582 cohorts were analyzed. The CRC patients with more advanced clinical stages in TCGA-COAD cohort, such as advanced pathological stage (*P* = 0.00022, Figure 3A), later T stage (*P* = 0.0032, Figure 3B), later M stage (*P* = 0.0042, Figure 3C), and later N stage (*P* = 0.0017, Figure 3D) had higher risk scores, indicating patients with low-risk scores have a better prognosis. Analogously, there were differences in the risk scores of different pathological stages (*P* = 0.018, Figure 3E) and different T stages (*P* = 0.0051, Figure 3F) in GSE39582 cohort, suggesting that risk scores were correlated with pathological stages and T stages. Besides, the risk score in M1 was not significantly different from M0 (*P* = 0.26, Figure 3G), while the risk score in N2/N3 was significantly higher than N0/N1 (*P* = 0.00045, Figure 3H). These results indicated that TALRGs signature has good clinical performance.

### Construction of a nomogram

**Figure 4:**
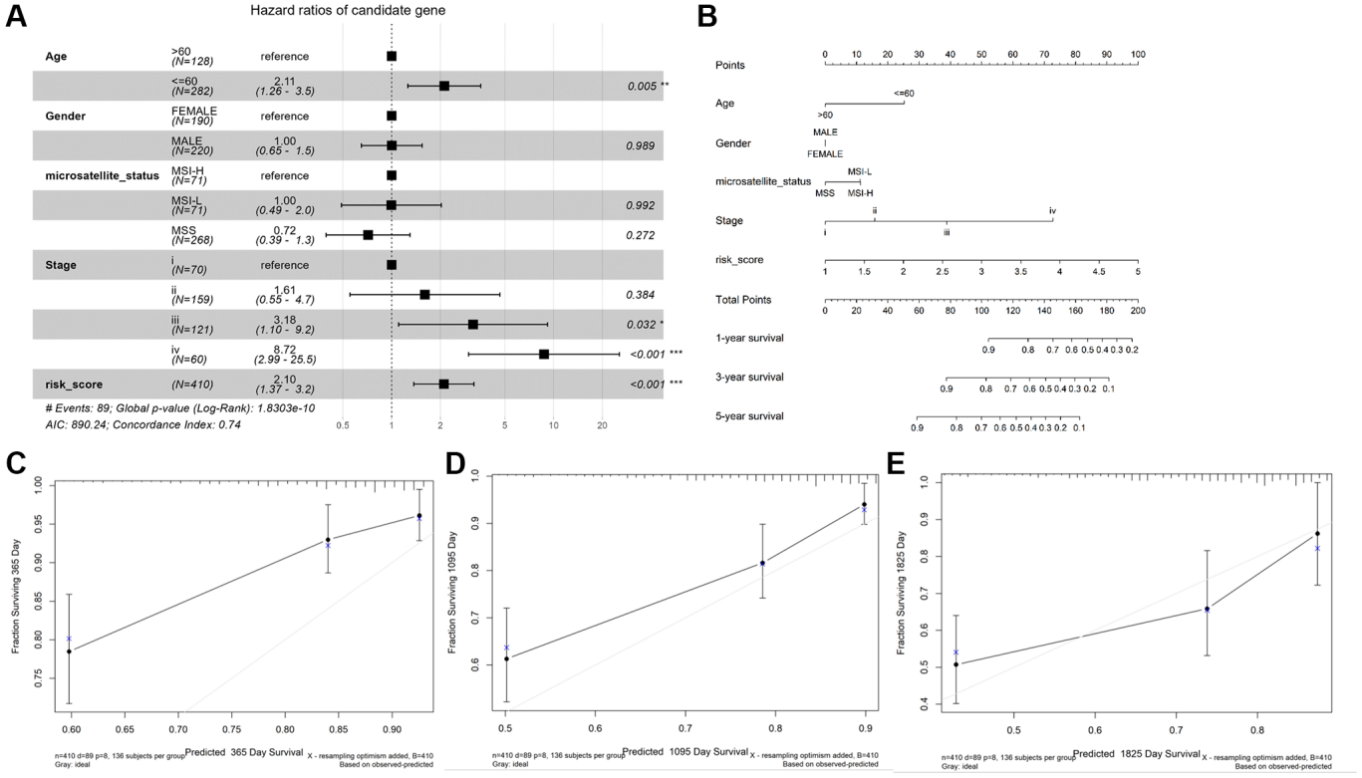
**Construction and validation of a nomogram.** (**A**) The forest map demonstrated the association between risk score, clinical characteristics, and OS. (**B**) Nomogram based on risk score and clinical characteristics in predicting 1-, 3-, and 5-years OS. Calibration curves were used to predict the probability of 1-year (**C**), 3-year (**D**), and 5-year (**E**) OS.

As shown in Figure 4A, age (≤ 60), stage III and IV, and risk score were observably associated with OS (*P* < 0.05), while gender, microsatellite status, stage I and II were not (*P* > 0.05). Then, we constructed a nomogram plot to ameliorate the accuracy of TALRGs signature and provided a quantitative and intuitive way to predict 1-, 3-, and 5-years survival probabilities for CRC patients (Figure 4B). We can determine the total score based on the individual score and thus predict the probability of survival of the patient at 1-, 3-, and 5-years. In addition, the calibration curves of the model showed that the predicted curves are close to the ideal curves, indicating that the nomogram has a good prediction performance (Figure 4C–4E).

### Immune infiltrate analysis and efficacy prediction of TALRGs signature

**Figure 5:**
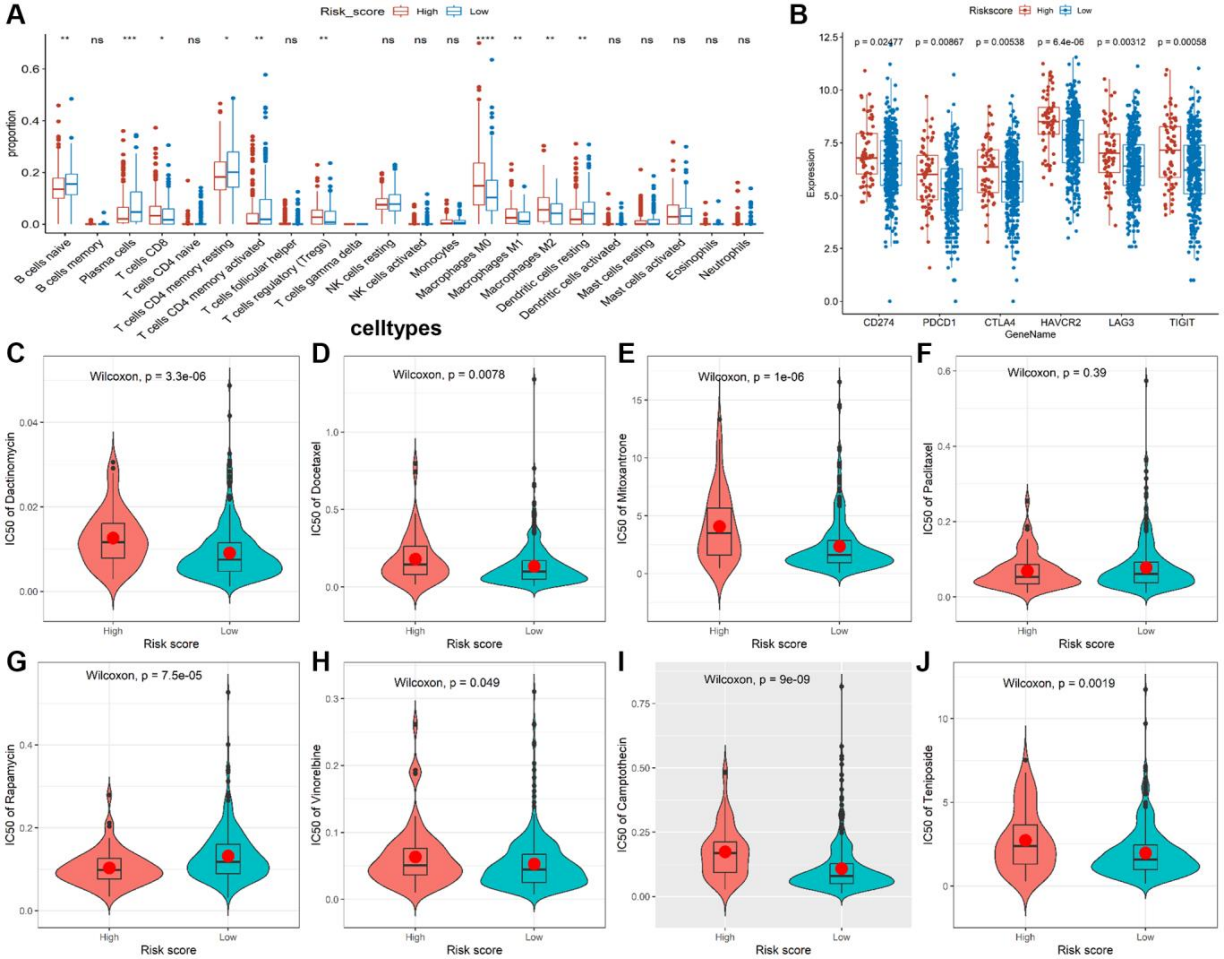
**Immune infiltrate analysis and efficacy prediction of TALRGs signature.** (**A**) The Wilcoxon test was applied to compare the proportions of immune cells between low- and high-risk groups. (**B**) Comparing the difference of the mRNA levels of immune checkpoints unpaired between the two risk groups using unpaired *t*-test. (**C**–**J**) Comparing the difference of IC50 value of chemotherapy drugs between the two risk groups using Wilcoxon test.

Given that the characteristics of tumor immune infiltrating cells are associated with tumor development and progression and may affect the prognosis of CRC, we used the CIBERSORT method to compare the immune infiltration of 22 immune cells in different risk groups. The results showed B cells, plasma cells, resting CD4+ T cells, activated CD4+ T cells, and resting dendritic cell (DC) cells were more abundant in the low-risk group, while CD8+ T cells, T regulatory cells (Tregs), and M0/M1/M2 macrophages were more abundant in the high-risk group (Figure 5A). In addition, we analyzed correlations between the risk model and CD274 (PDL1), PDCD1 (PD1), CTLA4, HAVCR2 (TIM3), LAG3, and TIGIT. We found observably higher expressions of all immune checkpoint molecules in the high-risk group (Figure 5B), indicating that immune function is suppressed in high-risk patients. Moreover, we discovered the IC_50_ of dactinomycin, docetaxel, mitoxantrone, vinorelbine, camptothecin, and teniposide was higher in the high-risk group, while the IC_50_ of rapamycin was higher in the low-risk group (Figure 5C–5J), suggesting that patients in the high-risk group are more resistant to most chemotherapy drugs.

### Mutation analysis of TALRGs signature

**Figure 6:**
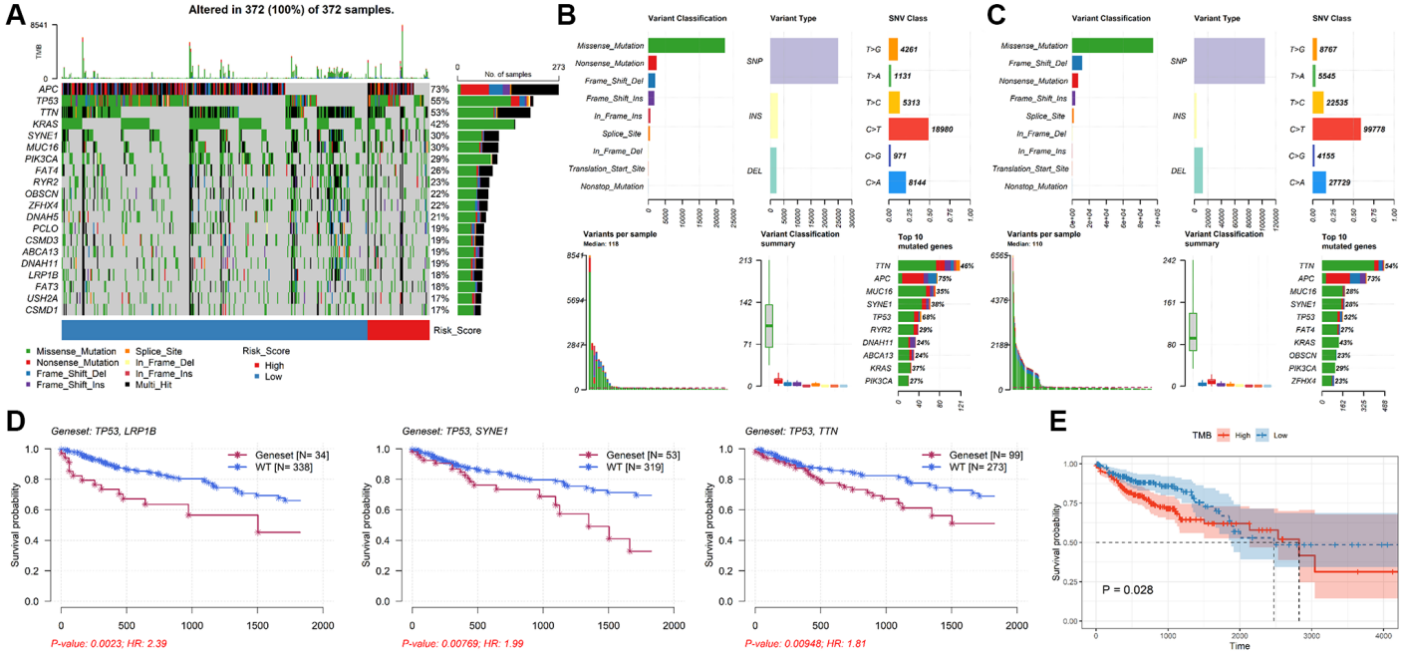
**Analysis of mutation characteristics of TALRGs signature.** (**A**) The mutation profiles of CRC patients. (**B**) The summary information of high-risk patients’ mutation profiles. (**C**) The summary information of low-risk patients’ mutation profiles. (**D**) Kaplan–Meier analysis of the two genes mutation status. (**E**) CRC patients with higher TMB values had poor OS.

Genetic alteration analysis showed that APC, TP53, TTN, KRAS, SYNE1, MUC16, PIK3CA, FAT4, RYR2 and OBSCN were the mutation rates of the top 10 genes in CRC patients (Figure 6A). The mutant profiles of high-risk and low-risk patients were shown in Supplementary Figure 2A, 2B, respectively. Besides, patients in the high-risk group had a higher median number of mutations than those in the low-risk group (118 vs. 110). In the high-risk group, single nucleotide polymorphism (SNP) was the most variant type, missense mutation was the most common SNP classification, and C > T was the main single nucleotide variants (SNV) class, and TTN, APC, MUC16, SYNE1, TP53, RYR2, DNAH11, ABCA13, KRAS, PIK32CA were the top 10 mutated genes in patients in the high-risk group (Figure 6B). Except for the top 10 mutated genes (TTN, APC, MUC16, SYNE1, TP53, FAT4, KRAS, OBSCN, PIK3CA, ZFHX4), the mutation profiles of the low-risk group were similar to that of the high-risk group (Figure 6C). Moreover, the mutation rate of TP53 in the high-risk group was significantly higher than in low-risk group (*P* = 0.0257, Supplementary Figure 2C). The mutation status of TP53 didn’t affect patients’ OS (*P* = 0.183, Supplementary Figure 2D), while TP53 combined with mutations in other genes (e.g. LRP18, SYNE1, TTN) significantly reduced survival time (Figure 6D). In addition, the median TMB value was 2.22/Mb in CRC patients (Supplementary Figure 2E), and there was no significant difference in TMB value between low-risk group and high-risk group (*P* = 0.8, Supplementary Figure 2F). However, patients with lower TMB had better prognosis (*P* = 0.028, Figure 6E).

### Effect of immunotherapy on prognosis

**Figure 7:**
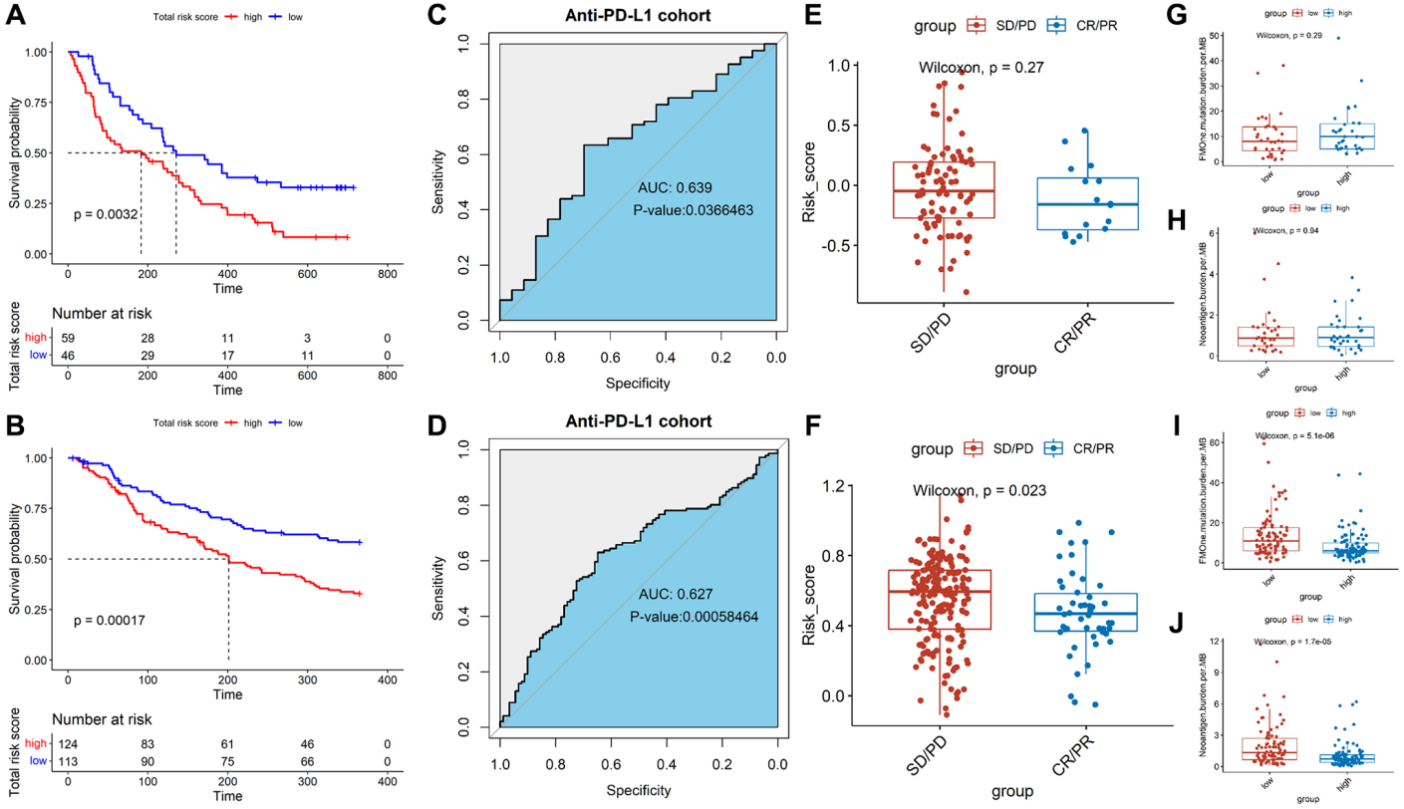
**Effect of immunotherapy on prognosis.** Kaplan–Meier analysis of the TALRGs signature in the platinum-treated cohort (**A**) and non-platinum-treated cohort (**B**). ROC curve analysis of anti-PD-L1 cohort in the platinum-treated cohort (**C**) and non-platinum-treated cohort (**D**). Comparing the difference of the risk score between responders group and non-responders group in the platinum-treated cohort (**E**) and non-platinum-treated cohort (**F**). Comparing the difference of the tumor mutation load and neoantigen load between low- and high-risk groups in the platinum-treated cohort (**G**, **H**) and non-platinum-treated cohort (**I**, **J**).

In order to evaluate the predictive effect of TALRGs signature on immunotherapy, we further validated constructed risk score model using the platinum-treated cohort and the non-platinum-treated cohort. The results exhibited patients with low-risk scores had significantly longer survival times (platinum-treated cohort: *P* = 0.0032, Figure 7A; non-platinum-treated cohort: *P* = 0.00017, Figure 7B). The AUCs of constructed model in anti-PD-L1 cohorts were 0.639 (platinum-treated cohort, Figure 7C) and 0.627 (non-platinum-treated cohort, Figure 7D), respectively. The clinical benefit of immunotherapy was not significant in patients receiving platinum therapy (*P* = 0.27, Figure 7E), while patients receiving non-platinum therapy marked clinical benefits from immunotherapy in the low-risk score group compared to those with a high-risk score group (*P* = 0.023, Figure 7F). In addition, higher tumor mutation load and higher neoantigen burden in platinum-treated patients was not significantly associated with risk score (*P* = 0.29, Figure 7G; *P* = 0.94, Figure 7H), while non-platinum-treated patients were associated with a low-risk score (*P* = 5.1e-06, Figure 7I; *P* = 1.7e-05, Figure 7J). These findings demonstrated that TALRGs signature is effective in predicting the prognosis of immunotherapy patients.

## DISCUSSION

Microorganisms play an important role in cancer occurrence, diagnosis, prognosis and treatment, especially CRC [20, 21]. Our previous research has identified 2 pathogenic microorganisms that can be used as biomarkers for COAD diagnosis and prediction [22]. However, due to technical limitations, the types of microorganisms in CRC and the depth of their role are not fully understood. Considering that microorganisms in tumors drive tumor progression by promoting immune tolerance [23], we applied bioinformatics tools for the first time to identify TALRGs based on LPS-related genes and T cells, and establish the prognostic risk model and nomogram for CRC. Then, the performance of TALRGs signature and its effect on immunotherapy were examined in multiple cohorts.

For this study, the prognostic 5-TALRGs genes HSPA1A, HSPB1, SERPINA1, SLC2A3, and TIMP1 were obtained. HSPA1A is a member of the heat shock protein group A (HSP70), which plays an important role in regulating the correct folding of proteins and maintaining protein homeostasis [24]. HSPA1A protects cell membrane integrity from bacterial LPS stimulation [25]. HSPA1A was down-regulated in CRC compared to adjacent nontumor tissues, which was consistent with other findings [26]. Additionally, Guan et al. [26] reported that increased HSPA1A in CRC was related to poor prognosis, which was consistent with our study. These findings indicated that HSPA1A may be related to progression rather than tumorigenesis of CRC. Besides, HSPB1 (also called human HSP27) as a member of HSPs, is involved in regulating molecular chaperones of cytoskeletal tissue [27] or stabilizing abnormally folded proteins to prevent aggregation [28]. LPS caused endothelial barrier dysfunction by promoting phosphorylation of HSPB1 [29]. HSPB1 is associated with the progression of CRC and can influence the response of CRC to different treatments [30]. SERPINA1 gene encodes alpha1-antitrypsin (A1AT), in which A1AT knockdown aggravates LPS-induced inflammation and cell death in human bronchial epithelial cells [31]. A1AT has been reported to bind complement components, which regulate T cell function [32]. Furthermore, SERPINA1 was reported to affect the CRC prognosis by regulating Th17 cells’ infiltration [33], which is considered as a biomarker of CRC diagnosis and prognosis [34]. SLC2A3, a member of the glucose transporter superfamily, was significantly up-regulated in LPS-induced human umbilical vein endothelial cells compared with control [35]. Gao and his team showed that elevated SLC2A3 is associated with poor survival of CRC [36], which is same as our study. Interestingly, SLC2A3 expression was positively related to CD4+ T cells and CD8+ T cells in COAD, respectively [36]. TIMP1 is a natural inhibitor of matrix metalloproteinases (MMPs), and increased LPS stimulation of macrophages during hepatitis C virus infection can lead to MMP/TIMP1 imbalance [37]. TIMP1 was markedly correlated with poor prognosis of CRC with its high expression [38]. Our results lead to a similar conclusion where TIMP1 is the prognostic biomarker for CRC. Meanwhile, TIMP1 expression was positively associated with CD8+ T cells in CRC [39]. From the findings above, the biological function of these 5-TALRGs were influenced by LPS and may affect the progression and immune response of CRC by modulating immune cells, especially T cells.

We constructed a prognostic model with high accuracy based on the combination of 5 prognostic TALRGs. Unlike other previous LPS-related prognostic models [40, 41], our 5-TALRGs signature contained the smallest number of genes and was relatively easy to apply clinically. As with other good prognostic signatures [42, 43], we also established a nomogram with better accuracy to contribute to predicting the prognosis of CRC using age, sex, microsatellite status, pathological stage, and risk score. Besides, TALRGs signature had a good performance in understanding immunological properties for CRC. For example, in the terms of immune cells infiltration, low-risk group had the higher infiltration of B cells, plasma cells, CD4+ T cells and DC cells, as well as lower infiltration of immunosuppressive cells, such as macrophages and Tregs. Studies have shown that the infiltration of B cells is associated with good prognosis of patients [44], which has anti-tumor effects for CRC [45]. Additionally, B cells are a major component and initiator of tertiary lymphoid structures (TLS), and tumors with mature TLS, high density of B cells and plasma cells typically have good clinical outcomes and respond to immunotherapy [46]. Besides, CD4+ T cells acted as an antitumor factor to provide clinical benefit to patients [47]. Furthermore, Tregs can promote chemotherapy resistance in CRC patients via FOXO1/CEBPB/NF-κB signaling pathway [48]. Therefore, the infiltration of different immune cells in different risk populations affects the response of colon cancer patients to immunotherapy.

Recent studies have shown that immunotherapy and chemotherapy can be used as adjunctive therapy for tumor patients [49], and have important implications for the treatment of colon cancer. In this study, risk stratification based on the TALRGs signature has been shown to help predict immune and chemotherapy responses. We found that patients with high-risk score had higher expression of immune checkpoint molecules and no sensitivity to multiple chemotherapy drugs of CRC, meaning that high-risk patients are more suitable for immunosuppressive therapy and chemotherapy drugs are more beneficial to low-risk patients. SNP is the most common variant type in the human genome [50]. Our study found SNP was the most variant type in both high-risk and low-risk patients, with the most missense mutations in the common SNP classification and C > T being the main SNV class, which is similar to previously study [17]. Missense mutation can be associated with a number of pathological conditions and can affect susceptibility to disease and drug treatment [51]. These findings suggested that the molecular mechanism of mutational effects can be revealed to understand the occurrence and development of CRC in order to improve the prognosis and clinical treatment of patients. In addition, immunotherapy efficacy is connected with TMB, usually MHC I molecules with high mutation load of tumors can produce more peptide neoantigens, which are recognized as “non-self”, thus triggering T cells to activate and kill tumor cells [52]. However, we found patients with lower TMB had better prognosis. The main mechanism of immune response of TMB is still being explored. More importantly, TALRGs signature was also significantly associated with OS in patients treated with anti-PD-L1. Similar to previous results [16], we speculated that patients with low-risk scores may be more sensitive to immune checkpoint inhibitor therapy. Briefly, based on the TALRGs signature, we can assign more reasonable treatment plans to patients, resulting in improving the survival rate of CRC patients.

## CONCLUSIONS

In conclusion, TALRGs signature constructed by the combination of T cells and LPS-related genes has a good performance in predicting prognosis and immune response of CRC, providing a promising strategy for guiding individualized therapy and improving prognosis prediction. This could aid in our understanding of the important role of microorganisms-mediated immune responses in the progression and prognosis of CRC.

## Supplementary Materials

Supplementary Figures

Supplementary Tables

## References

[1] Janney A, Powrie F, Mann EH. Host-microbiota maladaptation in colorectal cancer. Nature. 2020; 585:509–17. 10.1038/s41586-020-2729-3 32968260

[2] Keum N, Giovannucci E. Global burden of colorectal cancer: emerging trends, risk factors and prevention strategies. Nat Rev Gastroenterol Hepatol. 2019; 16:713–32. 10.1038/s41575-019-0189-8 31455888

[3] Johdi NA, Sukor NF. Colorectal Cancer Immunotherapy: Options and Strategies. Front Immunol. 2020; 11:1624. 10.3389/fimmu.2020.01624 33042104 PMC7530194

[4] Hossain MS, Karuniawati H, Jairoun AA, Urbi Z, Ooi J, John A, Lim YC, Kibria KMK, Mohiuddin AKM, Ming LC, Goh KW, Hadi MA. Colorectal Cancer: A Review of Carcinogenesis, Global Epidemiology, Current Challenges, Risk Factors, Preventive and Treatment Strategies. Cancers (Basel). 2022; 14:1732. 10.3390/cancers14071732 35406504 PMC8996939

[5] Wozniakova M, Skarda J, Raska M. The Role of Tumor Microenvironment and Immune Response in Colorectal Cancer Development and Prognosis. Pathol Oncol Res. 2022; 28:1610502. 10.3389/pore.2022.1610502 35936516 PMC9350736

[6] Formica V, Cereda V, Nardecchia A, Tesauro M, Roselli M. Immune reaction and colorectal cancer: friends or foes? World J Gastroenterol. 2014; 20:12407–19. 10.3748/wjg.v20.i35.12407 25253941 PMC4168074

[7] McCOY WC, Mason JM 3rd. Enterococcal endocarditis associated with carcinoma of the sigmoid; report of a case. J Med Assoc State Ala. 1951; 21:162–6. 14880846

[8] Hanahan D, Weinberg RA. The hallmarks of cancer. Cell. 2000; 100:57–70. 10.1016/s0092-8674(00)81683-9 10647931

[9] Park EM, Chelvanambi M, Bhutiani N, Kroemer G, Zitvogel L, Wargo JA. Targeting the gut and tumor microbiota in cancer. Nat Med. 2022; 28:690–703. 10.1038/s41591-022-01779-2 35440726

[10] Overacre-Delgoffe AE, Bumgarner HJ, Cillo AR, Burr AHP, Tometich JT, Bhattacharjee A, Bruno TC, Vignali DAA, Hand TW. Microbiota-specific T follicular helper cells drive tertiary lymphoid structures and anti-tumor immunity against colorectal cancer. Immunity. 2021; 54:2812–24.e4. 10.1016/j.immuni.2021.11.003 34861182 PMC8865366

[11] Nejman D, Livyatan I, Fuks G, Gavert N, Zwang Y, Geller LT, Rotter-Maskowitz A, Weiser R, Mallel G, Gigi E, Meltser A, Douglas GM, Kamer I, et al. The human tumor microbiome is composed of tumor type-specific intracellular bacteria. Science. 2020; 368:973–80. 10.1126/science.aay9189 32467386 PMC7757858

[12] Seifert L, Werba G, Tiwari S, Giao Ly NN, Alothman S, Alqunaibit D, Avanzi A, Barilla R, Daley D, Greco SH, Torres-Hernandez A, Pergamo M, Ochi A, et al. The necrosome promotes pancreatic oncogenesis via CXCL1 and Mincle-induced immune suppression. Nature. 2016; 532:245–9. 10.1038/nature17403 27049944 PMC4833566

[13] Vitiello GA, Cohen DJ, Miller G. Harnessing the Microbiome for Pancreatic Cancer Immunotherapy. Trends Cancer. 2019; 5:670–6. 10.1016/j.trecan.2019.10.005 31735286

[14] Das S, Shapiro B, Vucic EA, Vogt S, Bar-Sagi D. Tumor Cell-Derived IL1β Promotes Desmoplasia and Immune Suppression in Pancreatic Cancer. Cancer Res. 2020; 80:1088–101. 10.1158/0008-5472.CAN-19-2080 31915130 PMC7302116

[15] Mariathasan S, Turley SJ, Nickles D, Castiglioni A, Yuen K, Wang Y, Kadel EE III, Koeppen H, Astarita JL, Cubas R, Jhunjhunwala S, Banchereau R, Yang Y, et al. TGFβ attenuates tumour response to PD-L1 blockade by contributing to exclusion of T cells. Nature. 2018; 554:544–8. 10.1038/nature25501 29443960 PMC6028240

[16] Cao L, Li T, Ba Y, Chen E, Yang J, Zhang H. Exploring Immune-Related Prognostic Signatures in the Tumor Microenvironment of Colon Cancer. Front Genet. 2022; 13:801484. 10.3389/fgene.2022.801484 35281839 PMC8907673

[17] Cao L, Zhang S, Yao D, Ba Y, Weng Q, Yang J, Zhang H, Ren Y. Comparative analyses of the prognosis, tumor immune microenvironment, and drug treatment response between left-sided and right-sided colon cancer by integrating scRNA-seq and bulk RNA-seq data. Aging (Albany NY). 2023; 15:7098–123. 10.18632/aging.204894 37480572 PMC10415577

[18] Aran D, Looney AP, Liu L, Wu E, Fong V, Hsu A, Chak S, Naikawadi RP, Wolters PJ, Abate AR, Butte AJ, Bhattacharya M. Reference-based analysis of lung single-cell sequencing reveals a transitional profibrotic macrophage. Nat Immunol. 2019; 20:163–72. 10.1038/s41590-018-0276-y 30643263 PMC6340744

[19] Newman AM, Liu CL, Green MR, Gentles AJ, Feng W, Xu Y, Hoang CD, Diehn M, Alizadeh AA. Robust enumeration of cell subsets from tissue expression profiles. Nat Methods. 2015; 12:453–7. 10.1038/nmeth.3337 25822800 PMC4739640

[20] Jobin C. Precision medicine using microbiota. Science. 2018; 359:32–4. 10.1126/science.aar2946 29302001

[21] Cheng Y, Ling Z, Li L. The Intestinal Microbiota and Colorectal Cancer. Front Immunol. 2020; 11:615056. 10.3389/fimmu.2020.615056 33329610 PMC7734048

[22] Cao L, Wei S, Yin Z, Chen F, Ba Y, Weng Q, Zhang J, Zhang H. Identifying important microbial biomarkers for the diagnosis of colon cancer using a random forest approach. Heliyon. 2024; 10:e24713. 10.1016/j.heliyon.2024.e24713 38298638 PMC10828680

[23] Zhang X, Zhang K, Yan L, Wang P, Zhao F, Hu S. The role of toll-like receptors in immune tolerance induced by Helicobacter pylori infection. Helicobacter. 2023; 28:e13020. 10.1111/hel.13020 37691007

[24] Daugaard M, Rohde M, Jäättelä M. The heat shock protein 70 family: Highly homologous proteins with overlapping and distinct functions. FEBS Lett. 2007; 581:3702–10. 10.1016/j.febslet.2007.05.039 17544402

[25] Ding XZ, Feng XR, Borschel RH, Nikolich MP, Feng J, Li YS, Hoover DL. HSP-70 mitigates LPS/SKI-induced cell damage by increasing sphingosine kinase 1 (SK1). Prostaglandins Other Lipid Mediat. 2010; 92:1–7. 10.1016/j.prostaglandins.2009.12.006 20123033

[26] Guan Y, Zhu X, Liang J, Wei M, Huang S, Pan X. Upregulation of HSPA1A/HSPA1B/HSPA7 and Downregulation of HSPA9 Were Related to Poor Survival in Colon Cancer. Front Oncol. 2021; 11:749673. 10.3389/fonc.2021.749673 34765552 PMC8576338

[27] Carver JA, Rekas A, Thorn DC, Wilson MR. Small heat-shock proteins and clusterin: intra- and extracellular molecular chaperones with a common mechanism of action and function? IUBMB Life. 2003; 55:661–8. 10.1080/15216540310001640498 14769002

[28] Jakob U, Gaestel M, Engel K, Buchner J. Small heat shock proteins are molecular chaperones. J Biol Chem. 1993; 268:1517–20. 10.1016/S0021-9258(18)53882-5 8093612

[29] Hirano S, Rees RS, Yancy SL, Welsh MJ, Remick DG, Yamada T, Hata J, Gilmont RR. Endothelial barrier dysfunction caused by LPS correlates with phosphorylation of HSP27 in vivo. Cell Biol Toxicol. 2004; 20:1–14. 10.1023/b:cbto.0000021019.50889.aa 15119843

[30] Shimada T, Tsuruta M, Hasegawa H, Okabayashi K, Shigeta K, Ishida T, Asada Y, Suzumura H, Koishikawa K, Akimoto S, Kitagawa Y. Heat shock protein 27 knockdown using nucleotide-based therapies enhances sensitivity to 5-FU chemotherapy in SW480 human colon cancer cells. Oncol Rep. 2018; 39:1119–24. 10.3892/or.2018.6180 29328475

[31] He G, Yu W, Li H, Liu J, Tu Y, Kong D, Long Z, Liu R, Peng J, Wang Z, Liu P, Hai C, Yan W, Li W. Alpha-1 antitrypsin protects against phosgene-induced acute lung injury by activating the ID1-dependent anti-inflammatory response. Eur J Pharmacol. 2023; 957:176017. 10.1016/j.ejphar.2023.176017 37673367

[32] Dimeloe S, Rice LV, Chen H, Cheadle C, Raynes J, Pfeffer P, Lavender P, Richards DF, Nyon MP, McDonnell JM, Kemper C, Gooptu B, Hawrylowicz CM. Vitamin D (1,25(OH)_2_D3) induces α-1-antitrypsin synthesis by CD4^+^ T cells, which is required for 1,25(OH)_2_D3-driven IL-10. J Steroid Biochem Mol Biol. 2019; 189:1–9. 10.1016/j.jsbmb.2019.01.014 30690074 PMC6525112

[33] Li F, Zhou J, Li Z, Zhang L. Screening of immunosuppressive cells from colorectal adenocarcinoma and identification of prognostic markers. Biosci Rep. 2021; 41:BSR20203496. 10.1042/BSR20203496 33646276 PMC8024875

[34] Kwon CH, Park HJ, Choi JH, Lee JR, Kim HK, Jo HJ, Kim HS, Oh N, Song GA, Park DY. Snail and serpinA1 promote tumor progression and predict prognosis in colorectal cancer. Oncotarget. 2015; 6:20312–26. 10.18632/oncotarget.3964 26015410 PMC4653007

[35] Fu Q, Yu W, Fu S, Chen E, Zhang S, Liang TB. Screening and identification of key gene in sepsis development: Evidence from bioinformatics analysis. Medicine (Baltimore). 2020; 99:e20759. 10.1097/MD.0000000000020759 32629654 PMC7337576

[36] Gao H, Liang J, Duan J, Chen L, Li H, Zhen T, Zhang F, Dong Y, Shi H, Han A. A Prognosis Marker SLC2A3 Correlates With EMT and Immune Signature in Colorectal Cancer. Front Oncol. 2021; 11:638099. 10.3389/fonc.2021.638099 34211835 PMC8240412

[37] Fan C, Zhang X, Zhang P, Zhao J, Shen H, Zhang Y, Wu X, Jia Z, Wang Y. LPS stimulation during HCV infection induces MMP/TIMP1 imbalance in macrophages. J Med Microbiol. 2020; 69:759–66. 10.1099/jmm.0.001185 32242792 PMC7451043

[38] Qiu X, Quan G, Ou W, Wang P, Huang X, Li X, Shen Y, Yang W, Wang J, Wu X. Unraveling TIMP1: a multifaceted biomarker in colorectal cancer. Front Genet. 2023; 14:1265137. 10.3389/fgene.2023.1265137 37842645 PMC10570617

[39] Pan Z, Lin H, Fu Y, Zeng F, Gu F, Niu G, Fang J, Gu B. Identification of gene signatures associated with ulcerative colitis and the association with immune infiltrates in colon cancer. Front Immunol. 2023; 14:1086898. 10.3389/fimmu.2023.1086898 36742294 PMC9893113

[40] Yuan T, Zhang S, He S, Ma Y, Chen J, Gu J. Bacterial lipopolysaccharide related genes signature as potential biomarker for prognosis and immune treatment in gastric cancer. Sci Rep. 2023; 13:15916. 10.1038/s41598-023-43223-6 37741901 PMC10517958

[41] Che B, Zhang W, Li W, Tang K, Yin J, Liu M, Xu S, Huang T, Yu Y, Huang K, Peng Z, Zha C. Bacterial lipopolysaccharide-related genes are involved in the invasion and recurrence of prostate cancer and are related to immune escape based on bioinformatics analysis. Front Oncol. 2023; 13:1141191. 10.3389/fonc.2023.1141191 37188204 PMC10175693

[42] Cao L, Zhang S, Ba Y, Zhang H. Identification of m6A-related lncRNAs as prognostic signature within colon tumor immune microenvironment. Cancer Rep (Hoboken). 2023; 6:e1828. 10.1002/cnr2.1828 37178411 PMC10242659

[43] Cao L, Ba Y, Yang J, Zhang H. Exploring immune-related signatures for predicting immunotherapeutic responsiveness, prognosis, and diagnosis of patients with colon cancer. Aging (Albany NY). 2022; 14:5131–52. 10.18632/aging.204134 35748788 PMC9271306

[44] Federico L, McGrail DJ, Bentebibel SE, Haymaker C, Ravelli A, Forget MA, Karpinets T, Jiang P, Reuben A, Negrao MV, Li J, Khairullah R, Zhang J, et al. Distinct tumor-infiltrating lymphocyte landscapes are associated with clinical outcomes in localized non-small-cell lung cancer. Ann Oncol. 2022; 33:42–56. 10.1016/j.annonc.2021.09.021 34653632 PMC10019222

[45] Xu Y, Wei Z, Feng M, Zhu D, Mei S, Wu Z, Feng Q, Chang W, Ji M, Liu C, Zhu Y, Shen L, Yang F, et al. Tumor-infiltrated activated B cells suppress liver metastasis of colorectal cancers. Cell Rep. 2022; 40:111295. 10.1016/j.celrep.2022.111295 36044847

[46] Fridman WH, Meylan M, Petitprez F, Sun CM, Italiano A, Sautès-Fridman C. B cells and tertiary lymphoid structures as determinants of tumour immune contexture and clinical outcome. Nat Rev Clin Oncol. 2022; 19:441–57. 10.1038/s41571-022-00619-z 35365796

[47] Protti MP, De Monte L, Di Lullo G. Tumor antigen-specific CD4+ T cells in cancer immunity: from antigen identification to tumor prognosis and development of therapeutic strategies. Tissue Antigens. 2014; 83:237–46. 10.1111/tan.12329 24641502

[48] Wang D, Yang L, Yu W, Wu Q, Lian J, Li F, Liu S, Li A, He Z, Liu J, Sun Z, Yuan W, Zhang Y. Colorectal cancer cell-derived CCL20 recruits regulatory T cells to promote chemoresistance via FOXO1/CEBPB/NF-κB signaling. J Immunother Cancer. 2019; 7:215. 10.1186/s40425-019-0701-2 31395078 PMC6688336

[49] Gianni L, Valagussa P, Zambetti M, Moliterni A, Capri G, Bonadonna G. Adjuvant and neoadjuvant treatment of breast cancer. Semin Oncol. 2001; 28:13–29. 10.1016/s0093-7754(01)90042-9 11254864

[50] Lander ES, Linton LM, Birren B, Nusbaum C, Zody MC, Baldwin J, Devon K, Dewar K, Doyle M, FitzHugh W, Funke R, Gage D, Harris K, et al, and International Human Genome Sequencing Consortium. Initial sequencing and analysis of the human genome. Nature. 2001; 409:860–921. 10.1038/35057062 11237011

[51] Stefl S, Nishi H, Petukh M, Panchenko AR, Alexov E. Molecular mechanisms of disease-causing missense mutations. J Mol Biol. 2013; 425:3919–36. 10.1016/j.jmb.2013.07.014 23871686 PMC3796015

[52] Rizvi NA, Hellmann MD, Snyder A, Kvistborg P, Makarov V, Havel JJ, Lee W, Yuan J, Wong P, Ho TS, Miller ML, Rekhtman N, Moreira AL, et al. Cancer immunology. Mutational landscape determines sensitivity to PD-1 blockade in non-small cell lung cancer. Science. 2015; 348:124–8. 10.1126/science.aaa1348 25765070 PMC4993154

