# Supplementary material for: Exploration of bacterial lipopolysaccharide-related genes signature based on T cells for predicting prognosis in colorectal cancer
Source: Aging (Albany NY). 2024 Aug 6;16(15):11606–25. doi: 10.18632/aging.206041 (PMC11346792; doi:10.18632/aging.206041)
Supplement: Supplementary Figures [file aging-16-206041-s001.pdf]

SUPPLEMENTARY FIGURES

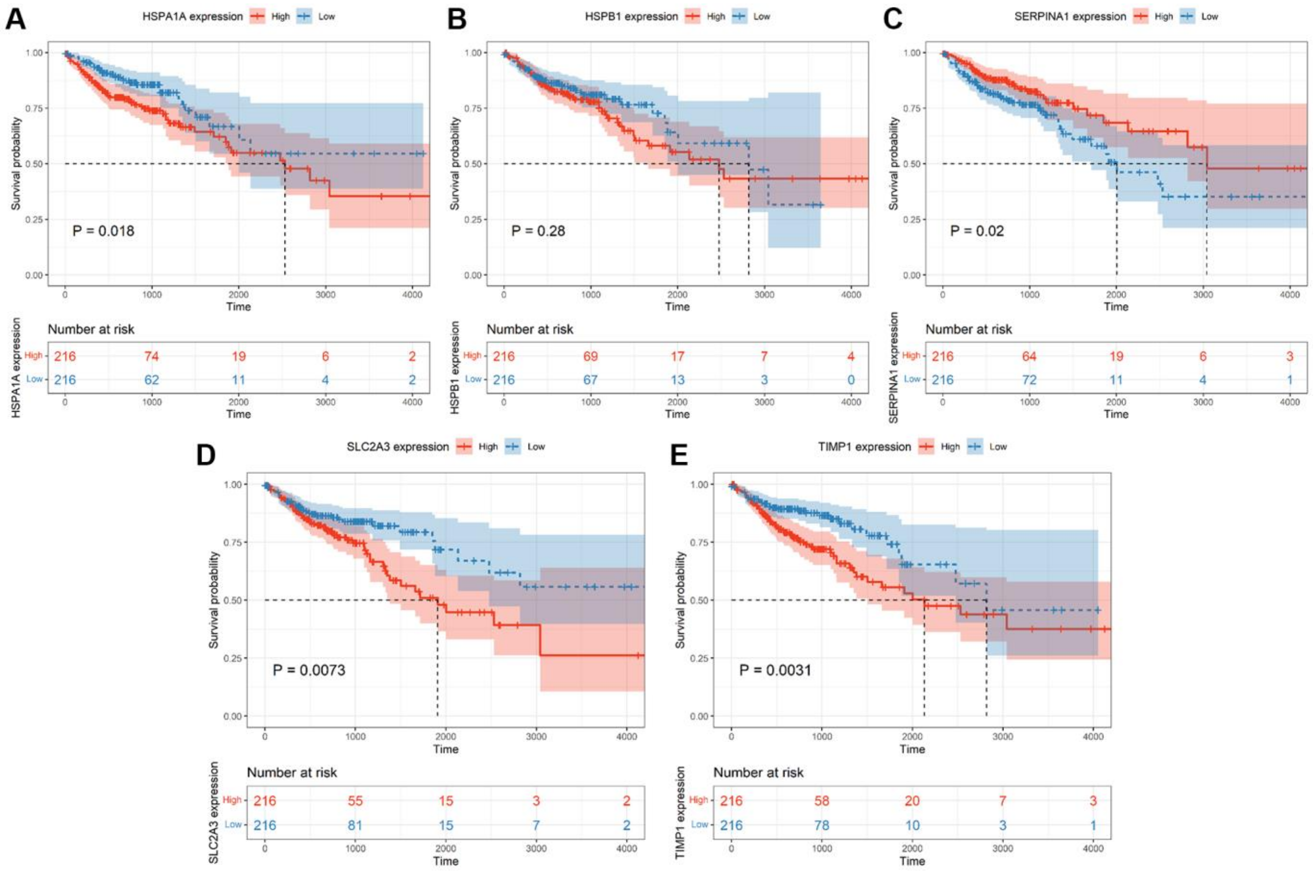

Supplementary Figure 1. Kaplan–Meier analysis of each TALRG mRNA expression, including HSPA1A, HSPB1, SERPINA1, SLC2A3, and TIMP1 (**A**–**E**).

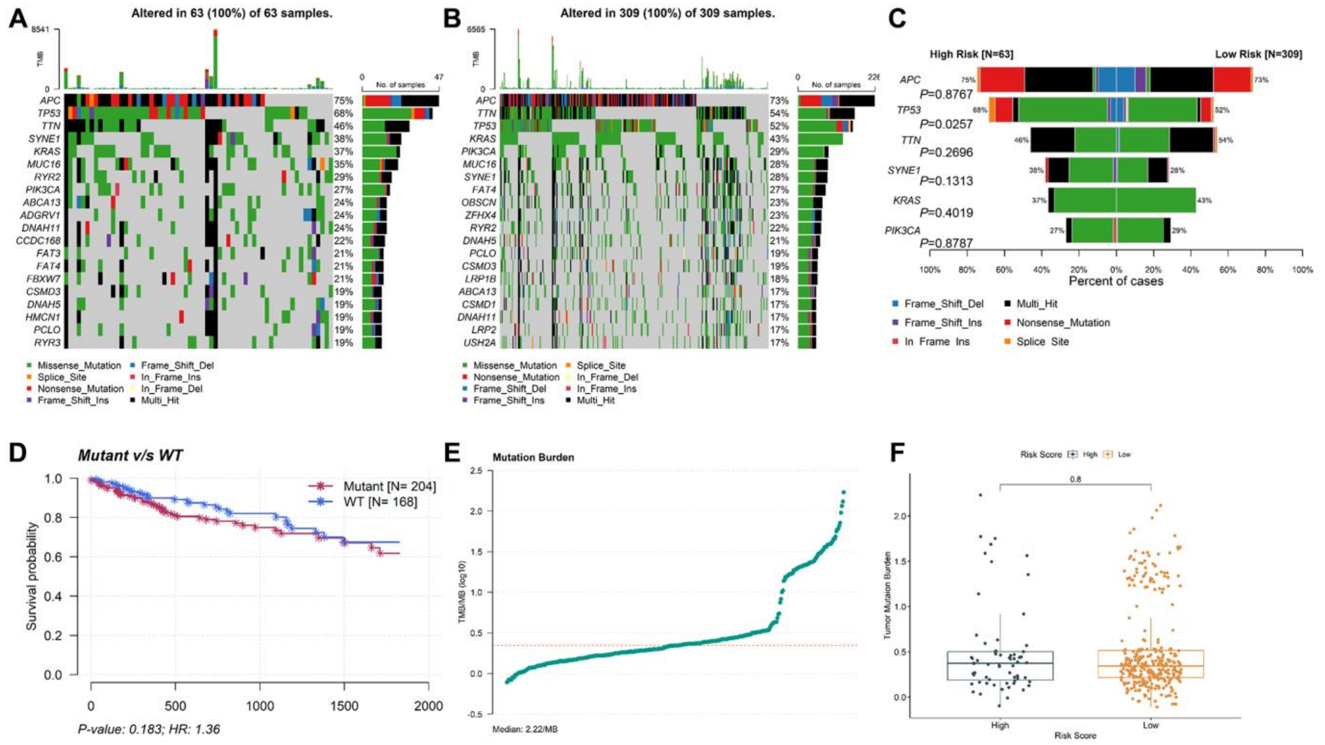

Supplementary Figure 2. Differences in mutation profiles between high- and low-risk groups. Waterfall plot of the top 20 mutated genes in high- (**A**) and low-risk groups (**B**). (**C**) Comparison of the mutation rate between the high- and low-risk groups. (**D**) Kaplan–Meier analysis of TP53 mutation status. (**E**) The TMB profiles of CRC patients. (**F**) Comparison of TMB values between the high- and low-risk groups.
