# Supplementary material for: Exploration of bacterial lipopolysaccharide-related genes signature based on T cells for predicting prognosis in colorectal cancer
Source: Aging (Albany NY). 2024 Aug 6;16(15):11606–25. doi: 10.18632/aging.206041 (PMC11346792; doi:10.18632/aging.206041)
Supplement: Supplementary Tables [file aging-16-206041-s002.pdf]

SUPPLEMENTARY TABLES

Supplementary Table 1. The detailed information of TCGA-COAD cohort.

	Tumor	Normal	Overall
	**(*N* = 432)**	**(*N* = 39)**	**(*N* = 471)**
**Factor (Gender)**			
Female	200 (46.3%)	20 (51.3%)	220 (46.7%)
Male	232 (53.7%)	19 (48.7%)	251 (53.3%)
**Factor (Age)**			
≤60	136 (31.5%)	9 (23.1%)	145 (30.8%)
>60	296 (68.5%)	30 (76.9%)	326 (69.2%)
**Factor (microsatellite_status)**			
	8 (1.9%)	3 (7.7%)	11 (2.3%)
Indeterminate	3 (0.7%)	0 (0%)	3 (0.6%)
MSI-H	72 (16.7%)	9 (23.1%)	81 (17.2%)
MSI-L	72 (16.7%)	6 (15.4%)	78 (16.6%)
MSS	277 (64.1%)	21 (53.8%)	298 (63.3%)
**Factor (Stage)**			
I	73 (16.9%)	4 (10.3%)	77 (16.3%)
II	166 (38.4%)	21 (53.8%)	187 (39.7%)
III	122 (28.2%)	6 (15.4%)	128 (27.2%)
IV	60 (13.9%)	7 (17.9%)	67 (14.2%)
Missing	11 (2.5%)	1 (2.6%)	12 (2.5%)
**Factor (T)**			
T1	11 (2.5%)	0 (0%)	11 (2.3%)
T2	75 (17.4%)	5 (12.8%)	80 (17.0%)
T3	296 (68.5%)	28 (71.8%)	324 (68.8%)
T4	49 (11.3%)	6 (15.4%)	55 (11.7%)
Missing	1 (0.2%)	0 (0%)	1 (0.2%)
**Factor (M)**			
M0	318 (73.6%)	25 (64.1%)	343 (72.8%)
M1	60 (13.9%)	7 (17.9%)	67 (14.2%)
MX	47 (10.9%)	6 (15.4%)	53 (11.3%)
Missing	7 (1.6%)	1 (2.6%)	8 (1.7%)
**Factor (N)**			
N0	254 (58.8%)	27 (69.2%)	281 (59.7%)
N1	100 (23.1%)	7 (17.9%)	107 (22.7%)
N2	78 (18.1%)	5 (12.8%)	83 (17.6%)

Supplementary Table 2. The detailed information of GSE39582 cohort.

	Alive	Death	Overall
	**(*N* = 369)**	**(*N* = 187)**	**(*N* = 556)**
**Factor (Sex)**			
Female	175 (47.4%)	74 (39.6%)	249 (44.8%)
Male	194 (52.6%)	113 (60.4%)	307 (55.2%)
**Factor (Age)**			
≤60	115 (31.2%)	42 (22.5%)	157 (28.2%)
>60	254 (68.8%)	145 (77.5%)	399 (71.8%)
**Factor (Stage)**			
0	3 (0.8%)	1 (0.5%)	4 (0.7%)
1	27 (7.3%)	5 (2.7%)	32 (5.8%)
2	183 (49.6%)	75 (40.1%)	258 (46.4%)
3	136 (36.9%)	67 (35.8%)	203 (36.5%)
4	20 (5.4%)	39 (20.9%)	59 (10.6%)
**Factor (T_stage)**			
N/A	8 (2.2%)	12 (6.4%)	20 (3.6%)
T0	1 (0.3%)	0 (0%)	1 (0.2%)
T1	10 (2.7%)	1 (0.5%)	11 (2.0%)
T2	36 (9.8%)	8 (4.3%)	44 (7.9%)
T3	249 (67.5%)	111 (59.4%)	360 (64.7%)
T4	63 (17.1%)	54 (28.9%)	117 (21.0%)
Tis	2 (0.5%)	1 (0.5%)	3 (0.5%)
**Factor (M_stage)**			
M0	340 (92.1%)	134 (71.7%)	474 (85.3%)
M1	20 (5.4%)	40 (21.4%)	60 (10.8%)
MX	1 (0.3%)	1 (0.5%)	2 (0.4%)
N/A	8 (2.2%)	12 (6.4%)	20 (3.6%)
**Factor (N_stage)**			
N/A	8 (2.2%)	12 (6.4%)	20 (3.6%)
N+	1 (0.3%)	5 (2.7%)	6 (1.1%)
N0	214 (58.0%)	81 (43.3%)	295 (53.1%)
N1	91 (24.7%)	40 (21.4%)	131 (23.6%)
N2	52 (14.1%)	46 (24.6%)	98 (17.6%)
N3	3 (0.8%)	3 (1.6%)	6 (1.1%)

Supplementary Table 3. Gene set from T cells comparison between the tumor and normal cells.

gene-ID	myAUC	avg_diff	power	avg_log2FC	pct.1	pct.2
JUNB	0.676	0.540385	0.352	0.779611	0.907	0.703
NR4A2	0.668	0.69728	0.336	1.005962	0.667	0.377
CD55	0.649	0.61498	0.298	0.887228	0.628	0.38
RPS26	0.352	−0.52224	0.296	−0.75344	0.897	0.929
CXCR4	0.641	0.455137	0.282	0.656624	0.767	0.547
BTG1	0.634	0.440019	0.268	0.634813	0.927	0.81
YPEL5	0.633	0.546502	0.266	0.788435	0.624	0.41
ZNF331	0.629	0.595867	0.258	0.859654	0.502	0.26
NR4A1	0.629	0.479171	0.258	0.691298	0.652	0.421
EZR	0.626	0.488399	0.252	0.704611	0.681	0.5
HERPUD1	0.624	0.425807	0.248	0.61431	0.764	0.606
S100A11	0.378	−1.0229	0.244	−1.47574	0.336	0.517
CD37	0.621	0.425842	0.242	0.614361	0.748	0.574
CD83	0.62	0.761335	0.24	1.098374	0.464	0.245
GTF2B	0.619	0.471917	0.238	0.680832	0.505	0.294
S100A6	0.381	−0.94463	0.238	−1.36281	0.477	0.639
LAPTM5	0.616	0.406139	0.232	0.585935	0.77	0.595
CD79A	0.615	0.698526	0.23	1.007759	0.5	0.315
CREM	0.613	0.5707	0.226	0.823346	0.492	0.29
SLC2A3	0.611	0.483525	0.222	0.697579	0.526	0.328
CD74	0.611	0.626941	0.222	0.904485	0.873	0.806
HLA-DPA1	0.606	0.488038	0.212	0.70409	0.591	0.424
HLA-DRB5	0.605	0.599606	0.21	0.865048	0.434	0.245
RHOH	0.605	0.479818	0.21	0.692231	0.51	0.329
HLA-DRB1	0.603	0.481777	0.206	0.695057	0.563	0.39
HIST1H4C	0.603	0.423851	0.206	0.611488	0.656	0.508
HLA-DRA	0.602	0.619545	0.204	0.893814	0.492	0.328
HLA-DQB1	0.601	0.527498	0.202	0.761018	0.455	0.27
HLA-DQA1	0.598	0.537681	0.196	0.77571	0.419	0.235
RBM38	0.593	0.439431	0.186	0.633965	0.291	0.108
LY9	0.593	0.524742	0.186	0.757042	0.347	0.17
PPDPF	0.411	−0.55898	0.178	−0.80644	0.786	0.829
CHMP1B	0.589	0.584106	0.178	0.842686	0.335	0.167
TCL1A	0.589	0.709733	0.178	1.023929	0.244	0.064
LGALS3	0.412	−0.82682	0.176	−1.19285	0.214	0.373
VPS37B	0.586	0.442933	0.172	0.639018	0.345	0.188
VPREB3	0.582	0.424238	0.164	0.612047	0.303	0.138
HSPB1	0.419	−0.55165	0.162	−0.79586	0.294	0.426
CSTB	0.422	−0.53586	0.156	−0.77308	0.338	0.465
NR4A3	0.578	0.413409	0.156	0.596423	0.264	0.111
GPR18	0.575	0.489747	0.15	0.706556	0.209	0.059
S100A4	0.426	−0.56929	0.148	−0.82132	0.357	0.473
HLA-DQA2	0.574	0.519909	0.148	0.75007	0.254	0.109

ANXA2	0.427	−0.61898	0.146	−0.893	0.189	0.321
TXN	0.428	−0.54125	0.144	−0.78085	0.3	0.418
S100A10	0.429	−0.76431	0.142	−1.10267	0.495	0.58
CST3	0.437	−0.83373	0.126	−1.20281	0.078	0.199
SELENOW	0.438	−0.40586	0.124	−0.58553	0.301	0.408
GSTP1	0.44	−0.4488	0.12	−0.64748	0.471	0.534
FCER2	0.559	0.41153	0.118	0.593712	0.179	0.062
TIMP1	0.442	−0.935	0.116	−1.34892	0.136	0.245
IGHM	0.552	0.506522	0.104	0.730756	0.192	0.089
LYZ	0.448	−1.18492	0.104	−1.70948	0.011	0.116
RAC1	0.448	−0.43494	0.104	−0.62748	0.31	0.384
GPX2	0.452	−0.82268	0.096	−1.18688	0.037	0.131
IFI27	0.455	−0.65298	0.09	−0.94206	0.055	0.144
CEBPD	0.455	−0.4333	0.09	−0.62512	0.099	0.188
HSPA6	0.455	−0.87952	0.09	−1.26888	0.054	0.142
KRT19	0.457	−0.82736	0.086	−1.19363	0.051	0.136
KRT18	0.457	−0.74114	0.086	−1.06924	0.104	0.186
LGALS1	0.458	−0.45727	0.084	−0.6597	0.248	0.318
KRT8	0.459	−0.62666	0.082	−0.90407	0.079	0.161
PHLDA2	0.459	−0.60937	0.082	−0.87913	0.06	0.139
MDK	0.461	−0.54889	0.078	−0.79189	0.036	0.112
HSPA1A	0.462	−0.59295	0.076	−0.85545	0.437	0.471
EPCAM	0.462	−0.5093	0.076	−0.73477	0.051	0.126
CTSB	0.463	−0.5349	0.074	−0.7717	0.177	0.245
CEACAM5	0.464	−0.66325	0.072	−0.95687	0.033	0.103
PERP	0.464	−0.43449	0.072	−0.62684	0.113	0.179
TSPAN8	0.465	−0.51586	0.07	−0.74423	0.038	0.109
CLDN3	0.465	−0.41945	0.07	−0.60514	0.068	0.139
IFITM3	0.466	−0.43279	0.068	−0.62439	0.244	0.293
HSPA1B	0.466	−0.47407	0.068	−0.68394	0.476	0.498
SERPINA1	0.467	−0.54867	0.066	−0.79156	0.037	0.103
S100A14	0.468	−0.41836	0.064	−0.60356	0.037	0.1
PIGR	0.473	−0.44955	0.054	−0.64857	0.057	0.11
IGHA1	0.481	−0.62369	0.038	−0.89979	0.197	0.228
GZMB	0.482	−0.45741	0.036	−0.65991	0.147	0.177
IGKC	0.483	−0.63964	0.034	−0.92281	0.236	0.262
MZB1	0.494	−0.44485	0.012	−0.64178	0.186	0.185
IGLL5	0.496	−0.46485	0.008	−0.67064	0.121	0.126
IGKV3-20	0.497	−0.42117	0.006	−0.60762	0.117	0.124
